# Rapid and repeated limb loss in a clade of scincid lizards

**DOI:** 10.1186/1471-2148-8-310

**Published:** 2008-11-11

**Authors:** Adam Skinner, Michael SY Lee, Mark N Hutchinson

**Affiliations:** 1 School of Earth and Environmental Sciences, The University of Adelaide, SA, 5005, Australia; 2 South Australian Museum, North Terrace, Adelaide, SA, 5000, Australia

## Abstract

**Background:**

The Australian scincid clade *Lerista *provides perhaps the best available model for studying limb reduction in squamates (lizards and snakes), comprising more than 75 species displaying a remarkable variety of digit configurations, from pentadactyl to entirely limbless conditions. We investigated the pattern and rate of limb reduction and loss in *Lerista*, employing a comprehensive phylogeny inferred from nucleotide sequences for a nuclear intron and six mitochondrial genes.

**Results:**

The inferred phylogeny reveals extraordinary evolutionary mutability of limb morphology in *Lerista*. Ancestral state reconstructions indicate at least ten independent reductions in the number of digits from a pentadactyl condition, with a further seven reductions proceeding independently from a tetradactyl condition derived from one of these reductions. Four independent losses of all digits are inferred, three from pentadactyl or tetradactyl conditions. These conclusions are not substantially affected by uncertainty in assumed rates of character state transition or the phylogeny. An estimated age of 13.4 million years for *Lerista *entails that limb reduction has occurred not only repeatedly, but also very rapidly. At the highest rate, complete loss of digits from a pentadactyl condition is estimated to have occurred within 3.6 million years.

**Conclusion:**

The exceptionally high frequency and rate of limb reduction inferred for *Lerista *emphasise the potential for rapid and substantial alteration of body form in squamates. An absence of compelling evidence for reversals of digit loss contrasts with a recent proposal that digits have been regained in some species of the gymnophthalmid clade *Bachia*, possibly reflecting an influence of differing environmental and genetic contexts on the evolution of limb morphology in these clades. Future study of the genetic, developmental, and ecological bases of limb reduction and loss in *Lerista *promises the elucidation of not only this phenomenon in squamates, but also the dramatic evolutionary transformations of body form that have produced the extraordinary diversity of multicellular organisms.

## Background

Limb reduction has occurred repeatedly during tetrapod evolution, affecting several morphologically and ecologically disparate clades (e.g., Aves, Lissamphibia, Mammalia, Squamata) [1,2]. Among these clades, Squamata (lizards and snakes) is regarded generally as an exemplary model for studying modes and causes of this potentially dramatic evolutionary phenomenon (e.g., [1-3]). At least 53 squamate lineages have been identified as having independently lost one or more bones of the fore- or hindlimb [3]. Many of these lineages are closely related and, accordingly, offer excellent material for comparative study. More significantly, a number of squamate clades include extant species displaying a range of intermediate states between pentadactyl and limbless conditions, affording the possibility of reconstructing patterns and rates of limb reduction and loss. Aside from their potential for elucidating the mechanisms and causes of limb reduction itself, such inferences of patterns and rates may contribute to an improved understanding of the substantial transformations of body form accompanying the emergence of higher taxa and, concomitantly, the relationship of macro- and microevolutionary phenomena (e.g., [4,5]).

Previous studies have examined several aspects of limb reduction in squamates. Wiens and Slingluff [4] presented an explicitly phylogenetic analysis of limb reduction and associated body elongation in the morphologically diverse clade Anguidae. Ancestral character state reconstructions provided evidence for three independent transitions to an elongate, functionally limbless body form, each of which was inferred to have occurred gradually over a period of more than 20 million years. Shapiro [6] and Shapiro *et al*. [7] demonstrated that digit loss in the scincid clade *Hemiergis *is not explained by simple truncation of a putative ancestral (i.e., pentadactyl) developmental program. Instead, structurally reduced limbs exhibit abbreviated embryonic expression of the gene *sonic hedgehog *and a presumably related decrease in the proliferation of limb mesenchyme. Wiens *et al*. [8] considered the roles of biogeography and competition in determining the frequency of transitions to an elongate, limb-reduced body form across squamates, proposing that discontinuity of habitats, dispersal limitation, and interspecific competition may be as significant as functional and developmental constraints in explaining the number of times such transitions have occurred. Kohlsdorf and Wagner [9] used phylogenetic methods to examine the evolution of limb morphology in the gymnophthalmid clade *Bachia*. Their analyses indicated that digits have been regained in some species of this clade and, accordingly, that the ability to develop digits may be preserved for an extended period after their loss. This conclusion challenges the traditional perspective that limb reduction is irreversible, and emphasises the need for further study of additional squamate clades to evaluate the generality of the above results.

Most recently, Brandley *et al*. [5] employed a time-calibrated phylogeny for 258 species to examine patterns in the evolution of an elongate, limbless body form across squamates. Their analyses revealed a consistent association of decreases in limb length, digit loss, and body elongation across ecologically and phylogenetically diverse squamate clades, indicating an influence of shared functional and developmental constraints on the evolution of body form. Transitions to a highly elongate, limbless body plan were estimated to have occurred over periods as brief as 16 million years, although intermediate morphologies (i.e., partial limb reduction, moderate body elongation) were inferred to persist for considerably greater lengths of time in some instances. This result intimates that such intermediate morphologies may be evolutionarily stable and, accordingly, are not necessarily transitory stages in an incomplete process of body elongation and limb loss. Although confirming the pervasiveness of structural reduction in the evolution of squamate limb morphology (at least 70 cases of digit loss were inferred), Brandley *et al*.'s [5] analyses identified at least six instances in which digits have been reacquired, supporting Kohlsdorf and Wagner's (2006) conclusion that limb reduction may be reversible.

While Brandley *et al*.'s [5] study is notable for its broad phylogenetic scope, the relatively sparse taxon sampling for several clades dictated (at least presently) by such an inclusive analysis potentially limits the resolution that can be attained in inferring the mode and tempo of evolutionary transitions. As Brandley *et al*. [5] note, denser taxon sampling may reveal that substantial limb reduction is possible over intervals considerably shorter than those indicated by their analyses. Similarly, inferred frequencies of digit loss and gain may be significantly altered by the inclusion of additional species; that Brandley *et al*.'s [5] analyses, which include only three of the 15 species considered by Kohlsdorf and Wagner [9], provide no evidence for reversals of limb reduction in *Bachia *serves to illustrate this point. Accordingly, detailed studies of comparatively recent clades are needed to establish general patterns and rates of squamate limb reduction and loss. A primary concern is whether the pattern indicated by analyses such as those of Wiens and Slingluff [4] and Brandley *et al*. [5], specifically, moderately frequent limb loss occurring over extended intervals (usually tens of millions of years) with occasional re-elaboration of limbs, accurately represents the evolution of body form in squamates. If analyses of more recent clades (which are less susceptible to sampling artefacts due to extinction) indicate consistently higher frequencies and rates of limb loss (or re-elaboration), there may be cause to query the perspective offered by these broader-scale studies. A number of diverse genera composed of species exhibiting varying degrees of limb reduction constitute ideal candidates for such analyses. The Australian scincid clade *Lerista *is pre-eminent among these, comprising more than 75 species displaying at least 20 distinct limb bone configurations, from that considered plesiomorphic for squamates (pentadactyl, with phalangeal formulae of 2.3.4.5.3 and 2.3.4.5.4 for the manus and pes, respectively) to entirely limbless [10,11].

Although *Lerista *has been considered the best available model for investigating squamate limb reduction [10,11], lack of a well-resolved phylogeny has impeded study of the pattern and mode of limb reduction and loss within the clade. Greer [10,11], who examined intra- and interspecific variation in phalangeal configurations for more than half of the species of *Lerista *then described, relied on an arrangement of observed configurations entailing the minimum amount of change between each in reconstructing sequences of phalanx loss, while noting that 'it would be most desirable to be able to order variation on the basis of a hypothesis of intrageneric relationships for *Lerista *based on characters independent of those under study, but unfortunately, this is not yet possible' [10]. More than two decades later, the phylogeny of *Lerista *remains largely obscure. A modest number of species groups have been diagnosed explicitly on the basis of proposed apomorphic character states, implying monophyly [12-14], however, relationships within and among these species groups are unknown. In this paper, we present the first comprehensive phylogeny for *Lerista*, inferred from nucleotide sequences for a nuclear intron and six mitochondrial genes. This phylogeny is employed in reconstructing ancestral digit configurations, which, in conjunction with estimates of absolute ages for nodes, provide insight into the pattern and rate of limb reduction within the clade.

## Results and Discussion

**Figure 1:**
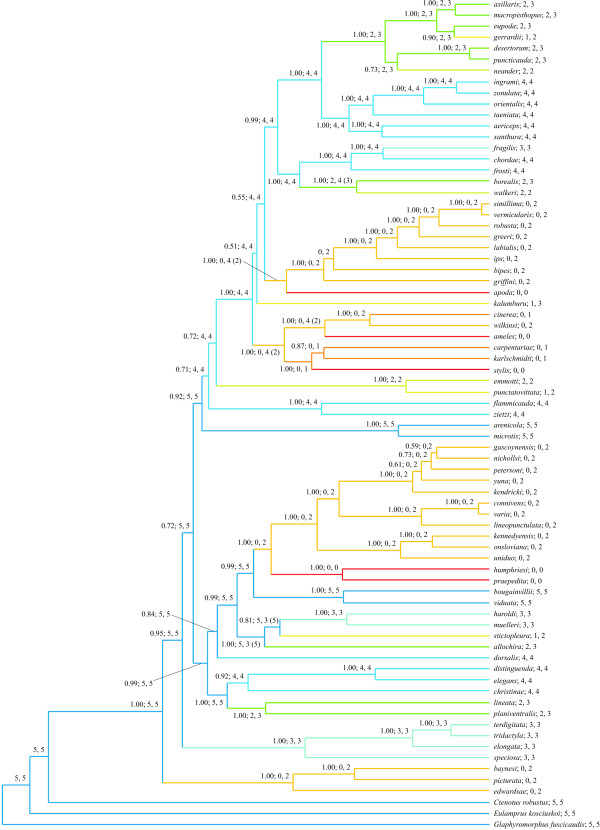
**Majority-rule consensus of 5000 trees sampled after attaining stationarity in a Bayesian analysis of nucleotide sequences for 72 species of *Lerista*.** Mean branch lengths from the Bayesian analysis have been modified via penalised likelihood rate smoothing to produce an ultrametric tree. Posterior probabilities ≥ 0.50 followed by maximum likelihood numbers of digits for the manus and pes are shown adjacent to internal nodes. Ancestral states for the manus were reconstructed assuming maximum likelihood rates of digit gain and loss; for the pes, ancestral states are those inferred assuming the maximum likelihood rate of digit loss and rates of digit gain of 0.020–0.025 (see text). Alternative numbers of pedal digits yielding a log-likelihood within two units of the optimal value (i.e., that for the maximum likelihood estimate) are presented in parentheses for ancestral digit configurations not observed among nominal species of *Lerista*. Modal digit configurations follow species names. Branches are coloured according to digit configuration: dark blue, 5, 5; light blue, 4, 4; light blue-green, 3, 3; green, 2, 3; light yellow-green, 2, 2; yellow, 1, 3 and 1, 2; light orange, 0, 2; dark orange, 0, 1; red, 0, 0.

Phylogenetic relationships were inferred from ATP synthetase-β subunit intron, 12S rRNA, 16S rRNA, and ND4 and adjacent tRNA-His, tRNA-Ser, and tRNA-Leu nucleotide sequences (2859 aligned sites) for 72 species of *Lerista *(*c*. 90% of those presently recognised) and three outgroup taxa (*Ctenotus robustus*, *Eulamprus kosciuskoi*, and *Glaphyromorphus fuscicaudis*). A majority-rule consensus of trees sampled in a Bayesian analysis of the combined sequence data (Figure 1) includes many significantly-supported clades (those associated with a posterior probability > 0.95). Several of these clades correspond with putative monophyletic groups recognised previously on the basis of morphology, including the *bipes *species group [13], the *nichollsi *species group (including *lineopunctulata*; see [13]), and the *orientalis *species group (excluding *muelleri*; see [14]). However, the majority of recovered relationships have never been hypothesised by earlier authors (e.g., the sister group relationship of *apoda *and the *bipes *species group, and of *praepedita *+ *humphriesi *and the *nichollsi *species group). The four species with well-developed, pentadactyl limbs (*arenicola*, *bougainvillii*, *microtis*, *viduata*) compose two distant clades, neither of which is positioned basally within *Lerista*. Those species in which limbs are greatly reduced (*carpentariae*, *cinerea*, *humphriesi*, *karlschmidti*, *praepedita*, *stylis*, *wilkinsi*) or absent (*ameles*, *apoda*) similarly are polyphyletic.

To investigate the pattern and rate of limb reduction within *Lerista*, we reconstructed numbers of digits for internal nodes assuming a Markov model of character evolution with separate parameters describing instantaneous rates of digit gain and loss. The number of digits for the manus and pes were considered independently. For the manus, the ratio of maximum likelihood rates of digit gain and loss (9.524 × 10^-5^) indicates that increases in the number of digits are far less probable than reductions. Maximum likelihood reconstructions of ancestral states imply 19 instances of digit loss with no reversals to an increased number of digits (Figure 1). All rates of digit gain not significantly less likely than the maximum likelihood estimate yield maximum likelihood ancestral states identical to or only insignificantly more likely than those reconstructed assuming the maximum likelihood rate (in comparing maximum likelihood and alternative states, log-likelihood differences of no more than two units were considered insignificant; see Methods).

For the pes, the estimated rate of digit gain exceeds that of digit loss (ratio of rates of gain and loss 2.056), with maximum likelihood reconstructions of ancestral states indicating 18 increases in the number of digits and 8 reductions (see Additional file 1). Although suggesting several reversals of limb reduction in *Lerista*, we regard this result as equivocal for two reasons. Firstly, a range of rates of digit gain for the pes are nearly as likely as the maximum likelihood estimate (0.394), and some of these rates yield ancestral state reconstructions implying no increases in the number of digits. Specifically, rates of 0.020–0.025 are not significantly less likely than the maximum likelihood rate and yield maximum likelihood ancestral states indicating 25 cases of digit loss but no instances in which digits are gained (Figure 1). Secondly, when considered in conjunction with states inferred for the manus, reconstructions of numbers of pedal digits (i.e., for the maximum likelihood rate of digit gain) imply implausible digit configurations for many internal nodes. In particular, configurations of either five or four digits for the manus and two digits for the pes are implied for the majority of basal nodes, however, neither is represented among extant species of *Lerista *(which never exhibit fewer digits for the pes than the manus [10,11]). Ancestral states reconstructed assuming rates of digit gain of 0.020–0.025, by contrast, imply implausible digit configurations for very few internal nodes. Moreover, in all instances, the ancestral states implying implausible digit configurations are not significantly more likely than alternative states implying configurations observed among extant species of *Lerista *(see Figure 1; this is not the case for ancestral states inferred under the maximum likelihood rate of digit gain). Thus, we are disinclined to conclude that limb reduction has been reversed in *Lerista*, preferring the ancestral states reconstructed assuming rates of pedal digit gain of 0.020–0.025 (however, see caveat below).

An examination of ancestral state reconstructions across 1000 trees (every fifth of 5000 trees) sampled after attaining stationarity in the Bayesian analysis indicates that the above conclusions are largely insensitive to uncertainty in the inferred phylogeny. For all nodes, maximum likelihood numbers of manual digits reconstructed for more than 90% of trees are consistent with (i.e., identical to or only insignificantly more likely than) those inferred assuming the Bayesian majority-rule consensus (see Figure 1), and reversals of limb reduction are inferred for only an insignificant proportion (less than 5%) of trees. Similar results are obtained when rates of digit gain considerably higher than those estimated by maximum likelihood are assumed. For example, a rate of 0.42, although significantly less likely than the (nearly invariably lower) maximum likelihood rates for 90% of trees, yields maximum likelihood ancestral states for 69 of 74 internal nodes consistent with those reconstructed assuming the Bayesian majority-rule consensus in more than 92% of trees, with more than 62% of trees exhibiting no reversals of limb reduction (note that branch lengths for post-stationarity trees are unscaled, so that rates are not immediately comparable to those for the Bayesian majority-rule consensus in Figure 1, in which branch lengths have been modified via penalised likelihood rate smoothing to produce an ultrametric tree; see Methods). Thus, the inferred ancestral states for the manus in Figure 1 are not substantially affected by uncertainty in tree topology, branch lengths, and rates of character state transition.

Ancestral state reconstructions for the pes are similarly stable when uncertainty in the phylogeny is considered. Assuming estimated rates of digit gain, maximum likelihood numbers of pedal digits for all nodes are consistent with those for the Bayesian majority-rule consensus (see Additional file 1) in more than 90% of trees. As for the Bayesian majority-rule consensus (see above), ancestral state reconstructions for nearly all trees (more than 95%) imply one or more instances of reversed limb reduction. Nonetheless, a range of rates of pedal digit gain only insignificantly lower than those estimated for a considerable proportion of trees yield ancestral state reconstructions very similar to those obtained for the Bayesian majority-rule consensus assuming rates of digit gain of 0.020–0.025 (entailing no increases in the number of digits; see Figure 1). A rate of 0.16, for example, is not significantly less likely than the estimated rates for 64% of trees and, for all except a single node, yields maximum likelihood ancestral states for more than 97% of trees that are consistent with the preferred ancestral states in Figure 1. In almost all cases, numbers of pedal digits reconstructed for the single exceptional node (the most recent shared ancestor of *christinae*, *distinguenda*, *elegans*, *lineata*, and *planiventralis*) entail no increases in the number of digits, and reversed limb reduction is implied in less than 5% of trees.

The phylogeny in Figure 1 thus implies extraordinary evolutionary mutability of limb morphology in *Lerista*. Although this conclusion does not depend on a preference for a specific set of ancestral states, the alternative reconstructions for the pes presented in Figure 1 and Additional file 1 evidently entail disparate patterns of limb evolution (repeated digit loss versus pervasive reacquisition of digits). As discussed above, there is no cogent statistical basis for favouring either of these reconstructions; ancestral states inferred assuming the maximum likelihood rate of digit gain (implying many reversals of limb reduction) are only insignificantly more likely than those reconstructed assuming rates of digit gain of 0.020–0.025 (entailing no reversals). However, when the very robust ancestral states for the manus are considered, a compelling argument can be made for accepting reconstructions for the pes implying no reversals of limb reduction. Whereas ancestral states entailing repeated re-elaboration of the pes imply digit configurations for many internal nodes differing fundamentally from those displayed by extant species of *Lerista*, ancestral digit configurations implied by reconstructions entailing no reversals of pedal digit loss are nearly invariably represented among observed phenotypes.

Although we prefer ancestral state reconstructions for the pes implying repeated, unreversed digit loss, we acknowledge that our results could be considered to indicate the reacquisition of pedal digits, and so provide further evidence for Kohlsdorf and Wagner's [9] proposal that limb reduction is potentially reversible. Perhaps significantly, the inferred age of *Lerista *(see below) is comparable to estimates of the period over which unexpressed developmental genes may preserve their functionality (0.5–6 million years [15]), rendering the re-elaboration of structurally reduced limbs at least plausible. Nonetheless, it should be noted that such re-elaboration would entail two instances in which a highly reduced pes (having two digits) has regained an entire or nearly entire complement of phalanges (*microtis *and *bougainvillii *exhibit phalangeal complements for the pes of 2.3.4.5.4 and 2.3.4.5.3, respectively). This contrasts with the inferred cases of digit reacquisition in *Bachia*, where species having putatively re-evolved digits exhibit unusual phalangeal complements (0.2.2.2.2 and 2.2.2.2.0 for the manus and pes, respectively), possibly reflecting the activation of a novel (i.e., non-ancestral) developmental pathway (Kohlsdorf and Wagner regarded this as additional evidence for the re-elaboration of limbs).

Allowing that pedal digit loss has not been reversed in *Lerista*, our analyses indicate a remarkably high frequency of limb reduction. Ancestral states reconstructed assuming the Bayesian majority-rule consensus, rates of digit gain of 0.020–0.025 for the pes, and maximum likelihood estimates for all other rates imply 11 independent reductions in the number of digits from a pentadactyl condition (ten if digit configurations for internal nodes observed among described species of *Lerista *are preferred; see Figure 1). A further seven reductions are inferred to have proceeded independently from a tetradactyl condition derived from one of these reductions (that in the most recent shared ancestor of *flammicauda *and the *orientalis *species group). Four independent losses of all manual and pedal digits are inferred, three from pentadactyl or tetradactyl conditions.

Assuming an age of 13.4 million years for *Lerista*, estimated using molecular dating methods (see Methods and Additional file 2), the loss of all digits from a pentadactyl condition in *ameles*, *apoda*, and *stylis *has occurred within a period of 11.8 million years. The most recent tetradactyl ancestor of *ameles *and *stylis *is no more than 9.7 million years old, while that of *apoda *is no more than 9.2 million years old. The highest rate of complete loss of digits from a pentadactyl or tetradactyl condition is inferred for *humphriesi *and *praepedita*; the most recent shared ancestor of these species (having a reconstructed digit configuration of no manual or pedal digits) is separated from an inferred pentadactyl ancestor by no more than 3.6 million years (it should be noted that limb loss could have occurred over only part of this interval, so that the actual rate of limb reduction may be substantially greater). This is considerably more rapid than the rate implied by Brandley *et al*.'s [5] estimate of the minimum interval over which limb loss has occurred in more than 20 squamate lineages (16 million years; see Background). As Brandley *et al*. [5] note, however, their estimates represent maximum values, and so may significantly overestimate the amount of time necessary for this radical evolutionary transition. Accordingly, it is at least conceivable that the higher rates of limb loss inferred here for *Lerista *are representative of those for squamates generally. This perspective suggests a pattern of body form evolution differing markedly from that envisaged by Wiens and Slingluff [4] for anguids (i.e., gradual change occurring over extended periods), and, if substantiated, would emphasise the significance of Brandley *et al*.'s [5] finding that intermediate phenotypes may persist for tens of millions of years.

**Figure 2:**
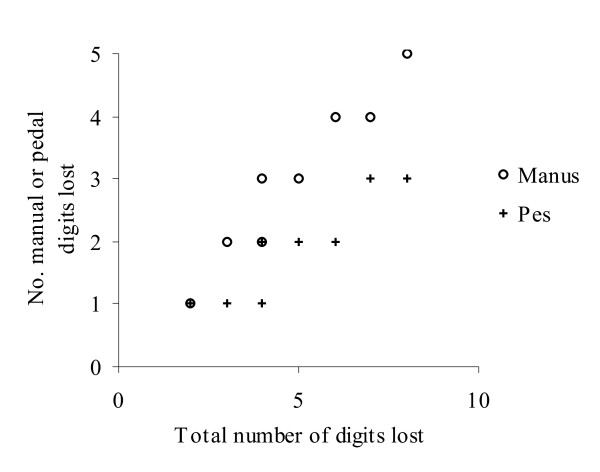
**Numbers of manual and pedal digits lost in all inferred reductions from a pentadactyl or tetradactyl condition. **Each point represents the number of manual or pedal digits initially lost by an inferred pentadactyl or tetradactyl ancestor; subsequent reductions (e.g., from a tridactyl condition produced as a consequence of the initial reduction) are not presented. 'Total number of digits lost' is the number of manual *and *pedal digits lost in an inferred reduction. Note the overlapping symbols at (2, 1) and (4, 2).

Almost invariably, species with greatly reduced limbs are separated from inferred pentadactyl or tetradactyl ancestors by very few nodes, so that, assuming limb reduction has not been saltational (i.e., that synchronous loss of several digits from the manus and pes has not occurred), reconstruction of complete sequences of digit loss for individual lineages is not possible. Nonetheless, if digit loss is presumed to proceed similarly across all (or at least many) lineages, general conclusions concerning the mode of limb reduction may be derived from a consideration of inferred losses for lineages exhibiting varying degrees of reduction. Thus, a plot of the numbers of manual and pedal digits lost in all reductions from a pentadactyl or tetradactyl condition versus the total number of (manual and pedal) digits lost (Figure 2) may be considered to indicate that in the initial stages of limb reduction rates of digit loss are similar for the manus and pes, however, as limb reduction progresses, manual digits are lost more readily than pedal digits. This is consistent with a more significant role of the hindlimb in limb-mediated locomotion and selection for some ability to employ limb-mediated locomotion (as an adjunct to undulatory locomotion, which becomes increasingly important as limb reduction and body elongation proceed) in limb-reduced species. Particular intermediate digit configurations, most conspicuously that of no digits for the manus and two digits for the pes, generally originate relatively rapidly and persist for extended periods. This pattern intimates that these configurations do not represent transitory stages in a continuing process of limb reduction and provides further evidence for adaptive retention of digits, consistent with Brandley *et al*.'s [5] proposal that intermediate phenotypes may be targets of selection.

## Conclusion

Many authors have noted the recurring evolution of an elongate, limb-reduced body form in squamates, however, even within this clade, the frequency and rate of limb reduction inferred here for *Lerista *are exceptional. Ancestral state reconstructions imply 27 instances of limb reduction (see Figure 1), almost as many as reported by Greer [3] for all remaining scincids, and nearly half the number of reductions inferred for all other squamates. An interval of no more than 3.6 million years for the complete loss of limbs in one lineage of *Lerista *is less than one fourth of Brandley *et al*.'s [5] minimum estimate of the time required for this substantial phenotypic transition. These results emphasise the potential for extensive alteration of body form in squamates over (geologically) brief periods. An absence of cogent evidence for reversals of digit loss in *Lerista *contrasts with Kohlsdorf and Wagner's [9] proposal that digits have been regained in some species of *Bachia*, possibly reflecting an influence of differing environmental and genetic contexts on the evolution of limb morphology in these clades. Future study of the genetic, developmental, and ecological bases of limb reduction and loss in *Lerista *promises the elucidation of not only this phenomenon in squamates, but also the dramatic evolutionary transformations of body form that have produced the extraordinary diversity of multicellular organisms.

## Methods

### Phylogenetic analysis

Specimen registration and collection locality data are provided in Additional file 3. ATP synthetase-β subunit intron, 12S rRNA, 16S rRNA, and ND4 and adjacent tRNA-His, tRNA-Ser, and tRNA-Leu fragments were amplified and sequenced as described by Skinner [16]. Alignment of ND4 sequences did not require the insertion of gaps and was straightforward. 12S, 16S, and tRNA sequences were initially aligned with Clustal X [17] assuming the default pairwise and multiple alignment parameter values. Adjustments to alignments were made with the aid of secondary-structure models [18-20]. ATP synthetase-β subunit intron sequences were aligned with Clustal X using the default settings. All sequences are deposited in GenBank (accession numbers, DQ915284, DQ915289, DQ915292, DQ915308, DQ915313, DQ915316, DQ915332, DQ915337, DQ915340, DQ915357, DQ915363, DQ915367, DQ915387, EF672754–EF673036).

Aligned sequences were partitioned according to locus and, for protein-encoding (i.e., ND4) sequences, codon position (the tRNAs were considered as a single partition, resulting in seven partitions; see Additional file 4). All partitions were analysed simultaneously using mixed-model Bayesian methods, implemented in MrBayes [21]. An appropriate nucleotide substitution model for each partition was selected on the basis of hierarchical likelihood-ratio tests, performed using Modeltest [22]. Four incrementally-heated Markov chains, initiated with random starting trees and default priors, were run for 10^7 ^generations, sampling every 1000^th ^generation. Parameter values for each specified model were estimated independently (i.e., parameter values were unlinked across partitions). The number of generations required to attain stationarity was estimated by examining cumulative posterior probabilities for clades, plotted using AWTY [23]. All trees sampled prior to attaining stationarity were discarded and the remaining trees used to compute a majority-rule consensus topology, branch lengths, and posterior probabilities for nodes. Parsimony and likelihood analyses yield results similar to those for the Bayesian analysis (see Additional files 5 and 6).

Penalised likelihood rate smoothing [24], performed with r8s [25], was employed to produce an ultrametric tree from the Bayesian majority-rule consensus (with mean branch lengths and an arbitrary age of 1.0 specified for the root node). This tree was assumed in inferring ancestral states (see below) and calculating absolute ages for nodes (see below and Additional file 2).

### Ancestral state reconstruction

Modal digit configurations for extant species of *Lerista *were collated from data in the literature, verified and augmented by our own observations of specimens in the South Australian Museum and Western Australian Museum. It should be noted that only those digits having one or more phalanges were counted; in some cases, species possess metacarpals and metatarsals for digits regarded here as absent. Maximum likelihood numbers of manual and pedal digits for internal nodes were determined independently using Mesquite [26], assuming the asymmetrical Markov k-state 2-parameter (AsymmMk) model of character evolution. The two parameters in this model describe instantaneous rates of forward and reverse transitions, corresponding in our analyses to rates of digit loss and gain. Inclusion of separate rate parameters for all possible transitions does not significantly improve model fit (the alternative models were compared by means of a likelihood-ratio test, performed using M. Pagel's program Multistate). Parsimony methods for inferring ancestral states may perform poorly where rates of character state transition are high (e.g., [27]) and are less amenable to statistical comparisons of alternative reconstructions than likelihood methods (see [28]), and, accordingly, were not employed.

The extent to which the ancestral state reconstructions depend on assumed rates of character state transition was assessed by comparing ancestral states inferred for a range of rates with those reconstructed assuming the maximum likelihood rates. Limb reduction is generally considered to be only exceedingly rarely (perhaps never) reversed (e.g., [3,10,11,29]; see, however, [5,9]) and we therefore focussed on rates of digit gain. Ancestral states were reconstructed for rates of digit gain representing all values not significantly less likely than the maximum likelihood estimate (according to likelihood-ratio tests) assuming estimated (i.e., optimal) rates of digit loss in each case.

As a means of evaluating the influence of uncertainty in inferred phylogenetic relationships and branch lengths, we reconstructed ancestral states across 1000 trees (every fifth of 5000 trees) sampled after attaining stationarity in the Bayesian analysis using the 'Trace Character Over Trees' option in Mesquite [26]. For each node in the Bayesian majority-rule consensus, numbers of manual and pedal digits were inferred for all trees containing that node; the variability of inferred states among trees provides a measure of the degree to which ancestral state reconstructions for the node concerned are affected by uncertainty in tree topology and branch lengths (see [30,31]). As this method may overestimate the confidence that can be placed in inferred ancestral states (because only trees incorporating the node of concern are examined [31]), the proportion of trees for which reconstructed ancestral states imply one or more reversals of limb reduction was also determined directly. A reversal was inferred where the greatest number of digits (or a higher number) observed among the set of species defined by a node was significantly less likely than the maximum likelihood state for that node (alternative states were compared on the basis of their contribution to the total likelihood for a tree, with differences of less than two log-likelihood units being considered insignificant; see [28]). Ancestral states were reconstructed across trees for a range of rates of digit gain as described above.

### Estimation of divergence times

Although no fossil record exists for *Lerista*, fossil scincids from the Oligo-Miocene to Pliocene limestone deposits of Riversleigh enabled the calibration of a phylogeny for lygosomines from which an absolute divergence time for two species of *Lerista *(*bipes *and *bougainvillii*) could be derived and used to calibrate the ultrametric tree in Figure 1. The lygosomine phylogeny was inferred from published 12S rRNA, 16S rRNA, and ND4 and adjacent tRNA-His, tRNA-Ser, and tRNA-Leu nucleotide sequences (2356 aligned sites) for 59 species. A majority-rule consensus of trees sampled in a Bayesian analysis of the combined sequence data is presented in Additional file 2. Penalised likelihood rate smoothing [24] was employed to produce an ultrametric tree from this consensus (with mean branch lengths and an arbitrary age of 10.0 specified for the root node) that was calibrated using the Riversleigh fossils. The calibrated phylogeny implies an absolute divergence time for *Lerista bipes *and *Lerista bougainvillii *of 12.1 million years, yielding an age of 13.4 million years for *Lerista *(see Additional file 2 for a more thorough discussion of the methods employed in estimating divergence times).

## Authors' contributions

AS, MSYL, and MNH conceived the study. AS collected the data and performed the analyses. AS, MSYL, and MNH wrote the paper. All authors have read and approved the manuscript.

## Supplementary Material

Additional file 1 Ancestral States Inferred Assuming Maximum Likelihood Rates of Digit Gain and Loss. Click here for file

Additional file 2 Estimation of the Absolute Age of *Lerista*. Click here for file

Additional file 3 Specimens Included in the Present Study. Click here for file

Additional file 4 Numbers of Aligned Sites, Variable Sites, and Unique Site Patterns for Partitions Employed in the Bayesian Analysis. Click here for file

Additional file 5 Parsimony Strict Consensus. Click here for file

Additional file 6 Maximum Likelihood Phylogeny. Click here for file
