# Supplementary material for: Rapid and repeated limb loss in a clade of scincid lizards
Source: BMC Evol Biol. 2008 Nov 11;8:310. doi: 10.1186/1471-2148-8-310 (PMC2596130; doi:10.1186/1471-2148-8-310)
Supplement: Additional file 1 — Ancestral States Inferred Assuming Maximum Likelihood Rates of Digit Gain and Loss. [file 1471-2148-8-310-S1.doc]

Bayesian majority-rule consensus (as in Figure 1 of our paper) with maximum likelihood numbers of digits for the manus and pes (inferred assuming maximum likelihood rates of digit gain and loss) shown adjacent to internal nodes.
