# Supplementary material for: Rapid and repeated limb loss in a clade of scincid lizards
Source: BMC Evol Biol. 2008 Nov 11;8:310. doi: 10.1186/1471-2148-8-310 (PMC2596130; doi:10.1186/1471-2148-8-310)
Supplement: Additional file 2 — Estimation of the Absolute Age of Lerista. [file 1471-2148-8-310-S2.doc]

Estimation of the Absolute Age of *Lerista*

Although no fossil record exists for *Lerista*, fossil scincids from the Oligo-Miocene to Pliocene limestone deposits of Riversleigh enabled the calibration of a phylogeny for lygosomines from which an absolute divergence time for two species of *Lerista* (*bipes* and *bougainvillii*) could be derived and used to calibrate the ultrametric tree presented in Figure 1 of our paper. Ideally, we would not have used this secondary calibration approach, however, nuclear intron sequence data were unavailable for virtually all species outside of *Lerista*; rather than estimating divergence times in a single analysis including only mitochondrial DNA sequences (or a substantial amount of missing data for many species), we decided to calibrate a phylogeny for *Lerista* based on mitochondrial DNA and nuclear intron sequences using an independently derived age estimate for the clade.

The lygosomine phylogeny was inferred from published 12S rRNA, 16S rRNA, and ND4 and adjacent tRNA-His, tRNA-Ser, and tRNA-Leu nucleotide sequences (2356 aligned sites) for 59 species (12S and 16S rRNA sequences for *Corucia zebrata* are from [1]; the remaining sequences are from [2] and [3]). A majority rule consensus of trees sampled in a Bayesian analysis of the combined sequence data is presented below. Penalised likelihood rate smoothing [4] was used to produce an ultrametric tree from this consensus (with mean branch lengths and an arbitrary age of 10.0 specified for the root node) that was assumed in calculating node ages.

The fossils employed in calibrating the lygosomine phylogeny include a partial mandible and several mandible fragments referred to the *Egernia* *striolata* and *Egernia frerei* species groups [5] and two partial dentaries described as *Tiliqua pusilla* [6], all present in deposits of early Middle Miocence age (*c*. 15 million years [7]). The synchronous occurrence of *Tiliqua pusilla* and species of *Egernia* establishes a minimum age for the node immediately below *Egernia whitii* and *Tiliqua adelaidensis* in the phylogeny below (labelled with an asterisk). Specifying an age of 15 million years for this node implies that *Lerista bipes* and *Lerista bougainvillii* diverged 12.1 million years ago, yielding an age of 13.4 million years for *Lerista*. This value is very similar to an estimate of 13.9 million years obtained assuming a rate of sequence divergence of 1.3% per million years [8] (the estimated age of 13.9 million years is based on a maximum uncorrected sequence divergence within *Lerista* of 18.114% for ND4 and the adjacent tRNAs).

Absolute age estimates for *Lerista* were also generated for 1000 trees (every sixth of 6000 trees) sampled after attaining stationarity in the Bayesian analysis of the lygosomine sequence data, permitting an assessment of uncertainty in our inferred age of 13.4 million years. All trees were converted to (uncalibrated) chronograms using penalised likelihood rate smoothing, assuming the optimal smoothing parameter for the majority-rule consensus and an arbitrary age of 10.0 for the root node. An age of 15 million years was then assigned to the node immediately below *Egernia whitii* and *Tiliqua adelaidensis*, scaling ages for the remaining nodes accordingly. Across trees, a mean age of 12.9 million years is obtained for the divergence of *Lerista bipes* and *Lerista bougainvillii*, with a 95% credible interval of 9.4-16.9 million years; corresponding values for the age of *Lerista* are 14.2 million years and 10.4-18.7 million years.

References

1. Honda M, Ota H, Kobayashi M, Hikida T: **Phylogenetic relationships of Australian skinks of the *Mabuya* group (Reptilia: Scincidae) inferred from mitochondrial DNA sequences.** *Genes Genet Syst* 1999, **74:** 135-139.

2. Reeder TW: **A phylogeny of the Australian *Sphenomorphus* group (Scincidae: Squamata) and the phylogenetic placement of the crocodile skinks (*Tribolonotus*): Bayesian approaches to assessing congruence and obtaining confidence in maximum likelihood inferred relationships.** *Mol Phylogenet Evol* 2003, **27:** 384-397.

3. Skinner A: **Phylogenetic relationships and rate of early diversification of Australian *Sphenomorphus* group scincids (Scincoidea, Squamata).** *Biol J Linn Soc* 2007, **92:** 347-366.

4. Sanderson MJ: **Estimating absolute rates of molecular evolution and divergence times: a penalized likelihood approach.** *Mol Biol Evol* 2002, **19:** 101-109.

5. Hutchinson MN: **Origins of the Australian scincid lizards: a preliminary report on the skinks of Riversleigh.** *Rec North Terr Mus (The Beagle)* 1992, **9:** 61-70.

6. Shea GM, Hutchinson MN: **A new species of lizard (*Tiliqua*) from the Miocene of Riversleigh, Queensland.** *Mem Queensl Mus* 1992, **32:** 303-310.

7. Archer M, Godthelp H, Hand SJ, Megirian D: **Fossil mammals of Riversleigh, northwestern Queensland: preliminary overview of biostratigraphy, correlation and environmental change.** *Aust Zool* 1989, **25:** 29-65.

8. Macey JR, Schulte II JA, Ananjeva NB, Larson A, Rastegar-Pouyani N, Shammakov M, Papenfuss TJ: **Phylogenetic relationships among agamid lizards of the *Laudakia caucasia* species group: testing hypotheses of biogeographic fragmentation and an area cladogram for the Iranian plateau.** *Mol Phylogenet Evol* 1998, **10:** 118-131.

Majority-rule consensus of trees sampled in a Bayesian analysis of lygosomine nucleotide sequences. Mean branch lengths from the Bayesian analysis have been modified via penalised likelihood rate smoothing to produce an ultrametric tree. A minimum age of 15 million years can be ascribed to the node indicated by an asterisk on the basis of fossil evidence.
