# Supplementary material for: Rapid and repeated limb loss in a clade of scincid lizards
Source: BMC Evol Biol. 2008 Nov 11;8:310. doi: 10.1186/1471-2148-8-310 (PMC2596130; doi:10.1186/1471-2148-8-310)
Supplement: Additional file 3 — Specimens Included in the Present Study. [file 1471-2148-8-310-S3.doc]

Specimens included in the present study. Abbreviations are: ABTC, Australian Biological Tissue Collection, South Australian Museum, Adelaide; AMS, Australian Museum, Sydney; NTM, Northern Territory Museum, Darwin; QM, Queensland Museum, Brisbane; SAMA, South Australian Museum, Adelaide; WAM, Western Australian Museum, Perth.

| Species | Registration number | Locality |
| --- | --- | --- |
| *aericeps* | SAMA R35983 | Mokari, SA |
| *allochira* | WAM R116698 | Vlaming Head, WA |
| *ameles* | SAMA R55815 | 35 km E Mt Surprise, Qld |
| *apoda* | WAM R114243 | Coulomb Point Nature Reserve, WA |
| *arenicola* | SAMA R50095 | Talia Beach, S Venus Bay, SA |
| *axillaris* | WAM R97212 | 21 km S Kalbarri, WA |
| *baynesi* | SAMA R26475 | Near Border Village, SA |
| *bipes* | SAMA R53899 | 63 km N Broome, WA |
| *borealis* | SAMA R51132 | El Questro Stn, WA |
| *bougainvillii* | SAMA R52630 | South Para Gorge, SA |
| *carpentariae* | NTM R14161 | Sir Edward Pellew Island, NT |
| *chordae* | SAMA R54482 | 22 km S Torrens Creek, Qld |
| *christinae* | WAM R115299 | Ellenbrook, WA |
| *cinerea* | SAMA R54520 | 3 km N Lolworth Homestead, Qld |
| *connivens* | SAMA R29286 | Carnarvon, WA |
| *desertorum* | WAM R92030 | 67 km SE Blue Robin Hill, WA |
| *distinguenda* | SAMA R20805 | Eyre Peninsula, SA |
| *dorsalis* | SAMA R48848 | Arcoona Stn, SA |
| *edwardsae* | SAMA R37941 | 10 km SE Moonabie Homestead, SA |
| *elegans* | WAM R90275 | 75 km SSW Karridale, WA |
| *elongata* | SAMA R42424 | Dog fence, near Coober Pedy, SA |
| *emmotti* | ABTC 31973 | Noonbah Stn, Qld |
| *eupoda* | WAM R135101 | Cue, WA |
| *flammicauda* | WAM R151177 | Tom Price, WA |
| *fragilis* | SAMA R55885 | 69 km S Alpha, Qld |
| *frosti* | SAMA R53312 | Trephina Gorge, NT |
| *gascoynensis* | WAM R116790 | Gascoyne Junction, WA |
| *gerrardii* | SAMA R22901 | Northampton, WA |
| *greeri*^1^ | WAM R108753 | 30 km SE Gordon Downs, WA |
| *greeri*^2^ | WAM R114424 | Derby, WA |
| *griffini* | NTM R22909 | Spirit Hills, Keep River, NT |
| *haroldi* | WAM R116653 | Gnaraloo Homestead, WA |
| *humphriesi* | WAM R116872 | Carrollgouda Well, WA |
| *ingrami* | QM J62430 | Cape Flattery, Qld |
| *ips* | WAM R131074 | Kiwirrkurra, WA |
| *kalumburu* | WAM R113949 | Carson Escarpment, WA |
| *karlschmidti* | NTM R23965 | Ramingining area, NT |
| *kendricki* | WAM R116264 | Kalbarri, WA |
| *kennedyensis* | WAM R99638 | Merlinleigh Homestead, WA |
| *labialis* | SAMA R48727 | 4 km W Mt Lindsay, SA |
| *lineata* | WAM R144983 | Jandakot Airport, WA |
| *lineopunctulata* | SAMA R 29778 | Scarborough Beach, Perth, WA |
| *macropisthopus* | SAMA R29294 | One Tree Point, Carnarvon, WA |
| *microtis* | WAM R90186 | 35 km NE Augusta, WA |
| *muelleri* | SAMA R54734 | Wirraminna Stn, SA |
| *neander* | WAM R104362 | 11 km SSW Capricorn Roadhouse, WA |
| *nichollsi* | AMS R123100 | Kalli Homestead, WA |
| *onsloviana* | WAM R116826 | Onslow, WA |
| *orientalis* | NTM R21731 | Litchfield National Park, NT |
| *petersoni* | WAM R99637 | Merlinleigh Homestead, WA |
| *picturata* | SAMA R22997 | 32 km S Norseman, WA |
| *planiventralis* | WAM R141463 | Faure Island, WA |
| *praepedita* | SAMA R29419 | Scarborough Beach, Perth, WA |
| *punctatovittata* | SAMA R42016 | Morgan Mail Road, SA |
| *puncticauda* | WAM R117169 | Queen Victoria Spring, WA |
| *robusta* | WAM R108783 | Cherrabun Homestead, WA |
| *simillima* | WAM R100874 | 6 km NW Fitzroy Crossing, WA |
| *speciosa* | SAMA R58029 | Sentinel Hill, SA |
| *stictopleura* | WAM R116825 | Mt Augustus, WA |
| *stylis* | NTM R21679 | Kakadu National Park, NT |
| *taeniata* | SAMA R32057 | 55 km S Immarna Siding, SA |
| *terdigitata* | SAMA R38568 | Middleback Ranges, SA |
| *tridactyla* | WAM R112650 | Ponier Rock, WA |
| *uniduo* | SAMA R29291 | One Tree Point, Carnarvon, WA |
| *varia*^2^ | SAMA R29388 | Nanga Dunes, WA |
| *varia*^1^ | WAM R141494 | Faure Island, WA |
| *vermicularis* | WAM R108778 | 6 km WNW Fitzroy Crossing, WA |
| *viduata* | WAM R116535 | Kundip, WA |
| *walkeri* | WAM R96954 | 29 km WSW Mt French, WA |
| *wilkinsi* | SAMA R55679 | 40 km S Torrens Creek, Qld |
| *xanthura* | WAM R135156 | Telfer Dome, WA |
| *yuna* | WAM R100846 | Yuna, WA |
| *zietzi* | WAM R114563 | 53 km WNW Newman, WA |
| *zonulata* | SAMA R55820 | Einasleigh Road, Qld |
|  |  |  |
| Outgroup |  |  |
| *Ctenotus robustus*^3^ | SAMA R36579 | Esdale, NSW |
| *Ctenotus robustus*^1^ | SAMA R36603 | Esdale, NSW |
| *Eulamprus kosciuskoi* | ABTC 1169 | Gloucester Falls, NSW |
| *Glaphyromorphus fuscicaudis* | ABTC 32148 | Mt Hartley, Qld |
|  |  |  |

^1^ATP synthetase-β subunit intron only; ^2^Mitochondrial genes only; ^3^Mitochondrial gene sequences from Reeder (2003) Mol Phylogenet Evol 27: 384-397.
