# Supplementary material for: Rapid and repeated limb loss in a clade of scincid lizards
Source: BMC Evol Biol. 2008 Nov 11;8:310. doi: 10.1186/1471-2148-8-310 (PMC2596130; doi:10.1186/1471-2148-8-310)
Supplement: Additional file 4 — Numbers of Aligned Sites, Variable Sites, and Unique Site Patterns for Partitions Employed in the Bayesian Analysis. [file 1471-2148-8-310-S4.doc]

Numbers of aligned sites, variable sites, and unique site patterns for partitions employed in the Bayesian analysis.

| Partition | Aligned sites | Variable sites | Unique site patterns |
| --- | --- | --- | --- |
|  |  |  |  |
| 12S rRNA | 902 | 425 | 501 |
| 16S rRNA | 548 | 216 | 252 |
| ND4, 1st codon position | 228 | 110 | 131 |
| ND4, 2nd codon position | 229 | 33 | 60 |
| ND4, 3rd codon position | 229 | 226 | 229 |
| tRNAs | 142 | 79 | 110 |
| ATP synthetase-β subunit | 581 | 276 | 405 |
|  |  |  |  |
