# Supplementary material for: Rapid and repeated limb loss in a clade of scincid lizards
Source: BMC Evol Biol. 2008 Nov 11;8:310. doi: 10.1186/1471-2148-8-310 (PMC2596130; doi:10.1186/1471-2148-8-310)
Supplement: Additional file 5 — Parsimony Strict Consensus. [file 1471-2148-8-310-S5.doc]

← Strict consensus of three trees obtained in a parsimony analysis of the nucleotide sequence data for *Lerista*, performed with PAUP* [1]. A heuristic search strategy was adopted, using random stepwise addition (100 replicates) and tree bisection and reconnection branch swapping. All sites were weighted equally. Non-parametric bootstrapping (100 replicates, each with 10 random stepwise addition replicates) was employed in assessing support for nodes; values greater than 50 are presented.

References

1. Swofford DL: *PAUP*: Phylogenetic Analysis Using Parsimony (* and other methods), Vers. 4.0.* Sunderland: Sinauer; 1999.
