# Supplementary material for: Rapid and repeated limb loss in a clade of scincid lizards
Source: BMC Evol Biol. 2008 Nov 11;8:310. doi: 10.1186/1471-2148-8-310 (PMC2596130; doi:10.1186/1471-2148-8-310)
Supplement: Additional file 6 — Maximum Likelihood Phylogeny. [file 1471-2148-8-310-S6.doc]

← Maximum likelihood phylogeny obtained using GARLI [1] assuming the GTR+I+Γ model and default search settings. Non-parametric bootstrapping (100 replicates) was employed in assessing support for nodes; values greater than 50 are presented.

References

1. Zwickl DJ: **Genetic algorithm approaches for the phylogenetic analysis of large biological sequence datasets under the maximum likelihood criterion.** *PhD thesis.* The University of Texas at Austin; 2006.
